# Three siblings with familial non-medullary thyroid carcinoma: a case series

**DOI:** 10.1186/s13256-016-0995-3

**Published:** 2016-08-02

**Authors:** Muhammad Owais Rashid, Naeemul Haq, Saad Farooq, Zareen Kiran, Sabeeh Siddique, Shahid Pervez, Najmul Islam

**Affiliations:** 1 Section of Endocrinology, Department of Medicine, Aga Khan University Hospital, Stadium Road, Karachi, Pakistan; 2 The Aga Khan University, Stadium Road, Karachi, Pakistan; 3 Department of Histopathology, The Aga Khan University, Karachi, Pakistan

**Keywords:** Familial, Non-medullary carcinoma, Thyroid, Case series

## Abstract

**Background:**

In 2015, thyroid carcinoma affected approximately 63,000 people in the USA, yet it remains one of the most treatable cancers. It is mainly classified into medullary and non-medullary types. Conventionally, medullary carcinoma was associated with heritability but increasing reports have now begun to associate non-medullary thyroid carcinoma with a genetic predisposition as well. It is important to identify a possible familial association in patients diagnosed with non-medullary thyroid carcinoma because these cancers behave more destructively than would otherwise be expected. Therefore, it is important to aggressively manage such patients and screening of close relatives might be justified. Our case series presents a diagnosis of familial, non-syndromic, non-medullary carcinoma of the thyroid gland in three brothers diagnosed over a span of 6 years.

**Case presentations:**

We report the history, signs and symptoms, laboratory results, imaging, and histopathology of the thyroid gland of three Pakistani brothers of 58 years, 55 years, and 52 years from Sindh with non-medullary thyroid carcinoma. Only Patients 1 and 3 had active complaints of swelling and pruritus, respectively, whereas Patient 2 was asymptomatic. Patients 2 and 3 had advanced disease at presentation with lymph node metastasis. All patients underwent a total thyroidectomy with Patients 2 and 3 requiring a neck dissection as well. No previous exposure to radiation was present in any of the patients. Their mother had died from adrenal carcinoma but also had a swelling in the front of her neck which was never investigated. All patients remained stable at follow-up.

**Conclusions:**

Non-medullary thyroid carcinoma is classically considered a sporadic condition. Our case report emphasizes a high index of suspicion, a detailed family history, and screening of first degree relatives when evaluating patients with non-medullary thyroid carcinoma to rule out familial cases which might behave more aggressively.

## Background

According to the American Cancer Society, in 2015 the number of people with thyroid cancer in the USA was approximately 63,000 and it caused 2000 deaths [1]. Approximately 1.1 % of people will be diagnosed with it at some point in their life, yet it remains one of the most treatable cancers with a median 5-year survival rate of 98 % [1]. Thyroid cancer is divided into two chief types: medullary, which arises from parafollicular C cells, and non-medullary, which arises from follicular epithelial cells. Non-medullary thyroid carcinoma (NMTC) includes papillary thyroid carcinoma, follicular thyroid carcinoma, Hürthle cell carcinoma, and anaplastic thyroid carcinoma and these make up 95 % of thyroid malignancies of which papillary carcinoma is the most common [2]. Although medullary carcinoma is traditionally associated with a genetic predisposition and a susceptibility gene, *RET*, has been identified, increasing evidence is now accumulating about the heritability of NMTC as well [2]. Familial NMTC (FNMTC) is defined as two or more first-degree relatives affected by thyroid cancer without another familial syndrome; this familial clustering has been reported in 3.5 to 10.0 % of cases [1, 3]. Heritability is usually in the form of syndromes such as familial adenomatous polyposis, Cowden syndrome, and Werner syndrome where the majority of tumors are in organs other than the thyroid. Non-syndromic FNMTC is a rare entity which most likely follows an autosomal dominant path with incomplete penetrance and variable expression [3].

## Case presentations

### Patient 1

**Fig. 1:**
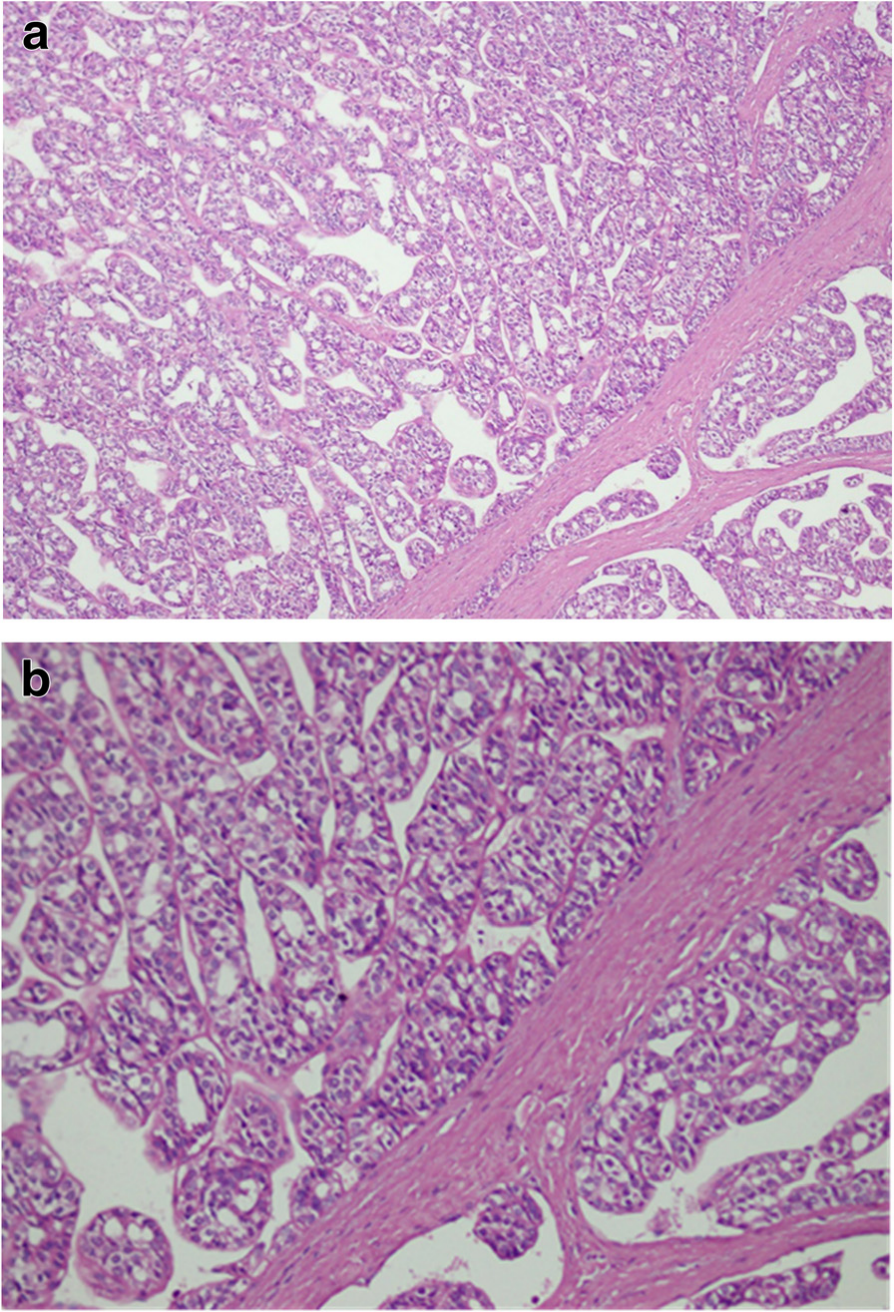
**a** Low power view of thyroid neoplasm with follicular arrangement. Invasion into capsular blood vessels can be seen in the *bottom right* of the image. **b** High power view of thyroid neoplasm with follicular arrangement. Nuclear features of papillary thyroid carcinoma are not visible

**Fig. 2:**
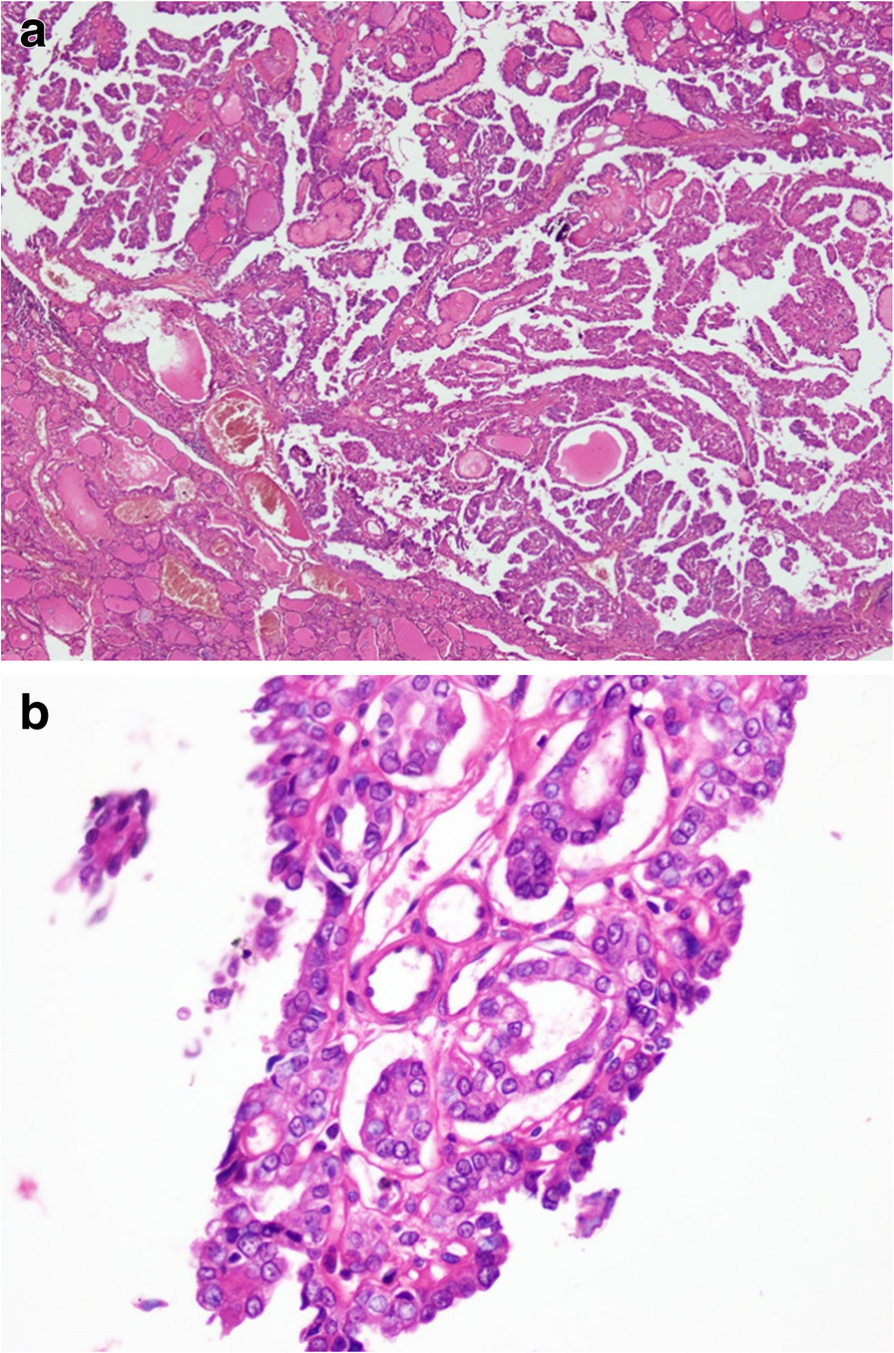
**a** Low power view of thyroid parenchyma showing a neoplastic lesion with papillary architecture. Scattered calcification is shown. **b** High power view of neoplastic cells exhibiting nuclear enlargement, crowding, clearing, grooves, and pseudoinclusions

A 58-year-old Muhajir Pakistani man presented to our surgery clinic with a swelling in his neck of 5 days’ duration, which he had noticed while shaving. On physical examination he had a left-sided thyroid nodule, approximately 6×4 cm with no lymphadenopathy. He was advised to have a thyroid function test, a thyroid ultrasound, and fine-needle aspiration (FNA) of the suspicious nodule. His laboratory investigations on follow-up showed thyroid-stimulating hormone (TSH) of 1.58 (0.4 to 4.2), thyroxine (T_4_) of 7.83 (5.1 to 14.1), and triiodothyronine (T_3_) of 1.99 (1.3 to 3.1). Ultrasonography of his thyroid gland revealed a multinodular goiter with largest nodule measuring 1.1×0.8 cm in right lobe and 2.3×1.2 cm in left lobe. Ultrasound-guided FNA of the left lobe of his thyroid showed a follicular lesion. According to American Thyroid Association (ATA) classification he was classified as an intermediate risk patient. A left lobectomy was planned for him but perioperative frozen section examination of the left lobe revealed a follicular carcinoma (Fig. 1a, b); therefore, a total thyroidectomy was performed and the tumor was completely resected. Surprisingly, histopathology of the thyroid specimen (right lobe) showed thyroid parenchyma infiltrated by a neoplastic lesion which had a papillary architecture (Fig. 2a, b). The papillary carcinoma measured 2×1.5×1 cm and was 0.2 cm away from the capsule; the follicular carcinoma measured 6×6 cm with no capsular breech. The cancer had a non-aggressive histology and no lymph nodes were involved. Well-formed papillary fronds were identified with prominent fibrovascular cores. In addition, psammoma bodies were also seen. After the surgery, he received 5550 MBq (150 mCi) radioactive iodine^131^ (RAI^131^) for remnant thyroid tissue ablation. His postoperative stimulated thyroglobulin levels were 19.10 (1.6 to 59.9) with a TSH of 39.12 (0.4 to 4.2). At 6-month follow-up, his stimulated thyroglobulin had increased to 56.56 ng/dl (1.6 to 59.9) with TSH of 94.66 (0.4 to 4.2). An ultrasound of his neck was normal and a whole body scan was negative; therefore, no distant metastasis was present. Considering the above laboratory values a second dose of 3700 MBq (100 mCi) iodine^131^ was given. He has been on regular follow-ups for the last 6 years without any evidence of recurrence.

### Patient 2

Patient 2 is a younger brother of Patient 1; Patient 2 is a 55-year-old man from Karachi, Pakistan who underwent a thyroid ultrasound for screening purposes although he was asymptomatic. No abnormality was noted on physical examination. His ultrasound showed an enlarged right lobe as compared to the contralateral side measuring 20.2×24.3×39.5 mm. At least three hypoechoic nodules with predominant solid components were seen in his right lobe with tiny calcification present within. The largest nodule measured 20.8×17.0 mm. Ultrasound-guided FNA revealed clusters and groups of follicular cells with architectural atypia, with a few of the cells forming papillary structures. Some nuclear grooving and intranuclear inclusions were seen with a group of Hürthle cells against a background of hemorrhage. He was classified as ATA intermediate risk and underwent total thyroidectomy with central neck dissection at another tertiary care facility. His histopathology report revealed a classic papillary thyroid carcinoma, 5.0 cm in diameter, with minimal extra thyroidal extension (right thyroid lobe). In addition, papillary microcarcinoma, Hürthle cell variant (0.5 cm), and follicular adenoma (left thyroid lobe) were reported as well with level VI lymph node micrometastasis. However, no capsular invasion was seen. He received postoperative 5550 MBq (150 mCi) RAI^131^ for remnant tissue ablation and was started on suppressive thyroid hormone replacement. He has been in follow-up for 3 years with no evidence of residual or recurrent disease.

### Patient 3

**Fig. 3:**
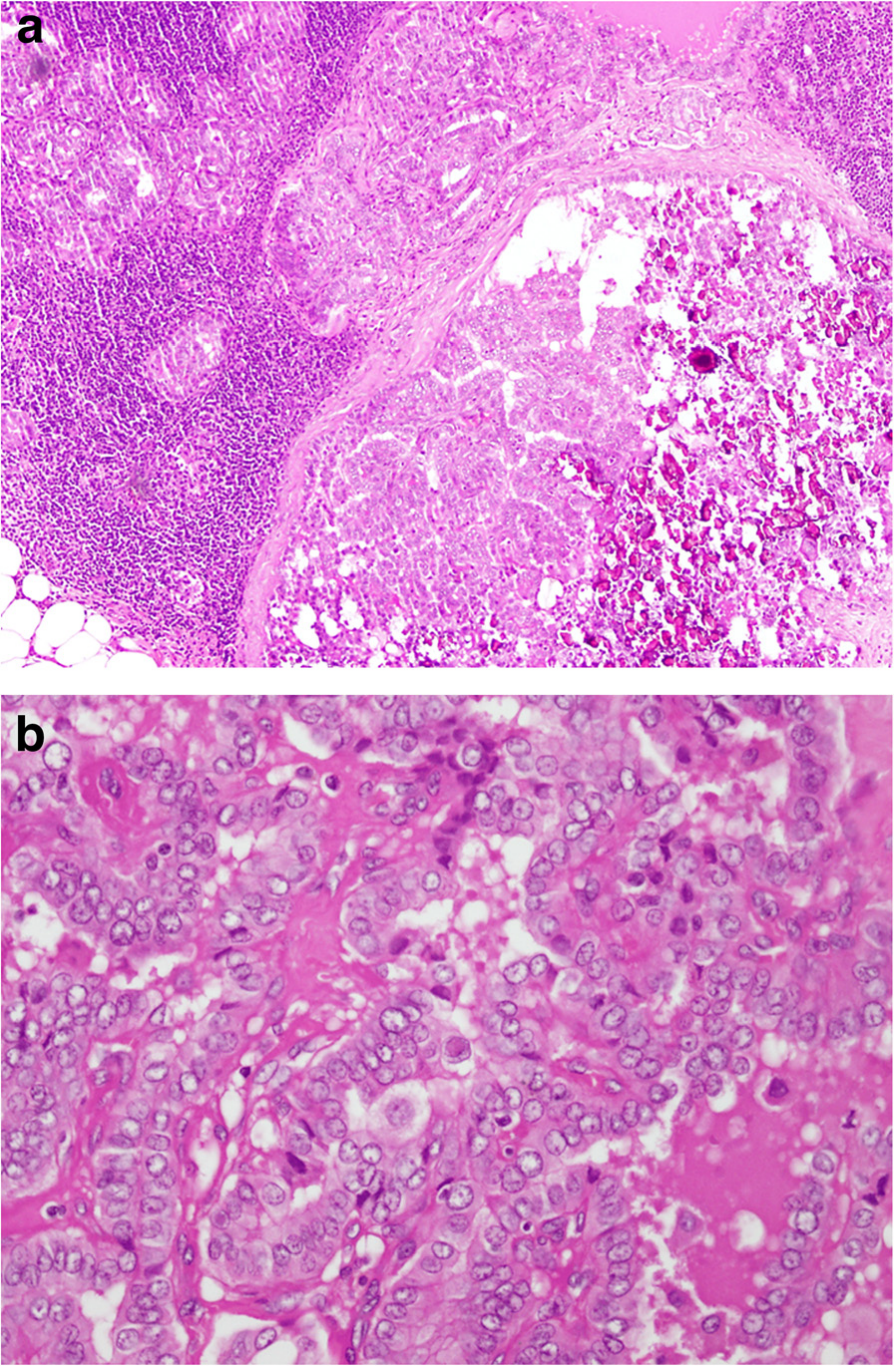
**a** Lymph node showing metastatic deposits. **b** Tumor with intranuclear inclusions

Patient 3 is the youngest brother of the family; Patient 3 is a 52-year-old Pakistani man who visited an endocrinologist in another hospital with the complaint of chronic urticaria. He had no other symptoms and had a normal physical examination. He was advised to have thyroid antibody tests which showed anti-thyroid peroxidase (TPO) of 695.10 (normally less than 35) and anti-thyroglobulin of 29.50 (normally less than 40). A thyroid function test showed TSH of 1.80 (0.4 to 4.2), T_4_ of 8.80 (4.6 to 10.5), and T_3_ of 2.01 (1.23 to 3). Considering the strong family history of papillary thyroid carcinoma, he was advised to have a thyroid ultrasound which showed a multinodular goiter. Fine-needle aspiration cytology (FNAC) revealed papillary carcinoma of the thyroid. A few clinically suspicious lymph nodes were also present bilaterally and he was classified as a high risk patient according to ATA guidelines. He underwent total thyroidectomy with bilateral selective neck dissection: level II, III, IV and VI. Histopathology confirmed papillary carcinoma, classic variant, which was 7×5.5×3 cm with capsular invasion and lymph node metastasis to level II, II, IV and IV bilaterally with no distant metastasis (Fig. 3a, b). He received postoperative 6660 MBq (180 mCi) RAI^131^ for remnant thyroid tissue ablation; he was started on suppressive thyroid hormone therapy. His follow-up ultrasound at 6 months showed a 14×11 mm heterogeneous area in his right paratracheal region with few lymph nodes and preserved hilum. The largest lymph node was on the right side and measured 15×5 mm. His thyroglobulin level was 46 ng/dl (1.6 to 59.9) whereas a whole body RAI^131^ scan was negative for residual disease. He underwent a positron emission tomography (PET) scan which showed a hypermetabolic, 8 mm right level II node: standardized uptake value (SUV) of 5.2. He therefore underwent a second surgery for residual disease and right-sided neck dissection in 2015. Histopathology showed metastatic lymph nodes. He has kept regular follow-ups for 2 years.

## Discussion

To the best of our knowledge this is the first case series reported of familial non-medullary carcinoma of the thyroid from Pakistan. Because the number of affected patients was more than two it is highly unlikely that the NMTC was due to sporadic mutations. No specific gene has been associated with heritability; therefore, no genetic testing is available to check for the specific gene. Therefore, clinicians have to rely on a strong family history of this variant of thyroid cancer to diagnose familial cases. In our case series, three brothers were affected; Charkes stated that when three or more family members are affected the probability of this due to sporadic mutations is less than 6 %; thus, we believe that FNMTC in our patients due to sporadic mutations is highly unlikely [4]. Although some researchers argue that familial clustering could be due to environmental exposure and bias due to more aggressive screening in asymptomatic family members, increasing evidence is now accumulating on the hereditability of NMTC. In our study, Patient 1 had a follicular carcinoma in the left and papillary carcinoma in the right lobe, respectively. In addition, Patient 2 had a classic papillary thyroid carcinoma along with papillary microcarcinoma, Hürthle cell variant, and follicular adenoma (left thyroid lobe), suggesting similar genetic mutations in the pathogenesis of FNMTC. As yet, the underlying genetic mutation involved in FNMTC has not been identified, although it has been suggested that FNMTC is a polygenic cancer syndrome as several susceptibility genes and candidate chromosomal loci have been reported [5–7].

All three of our patients had minimal symptoms but advanced disease at presentation. Lymph nodes metastasis was seen in Patients 2 and 3 whereas capsular invasion was present in Patient 3 only. This aggressive picture is supported by a meta-analysis by Wang *et al*. which showed that FNMTC is more aggressive at presentation with a higher degree of recurrence due to increased multifocality, extrathyroid invasion, bilateral presentation, and lymph node involvement and is associated with less disease-free survival as compared to sporadic NMTC [8]. It is also associated with anticipation, widespread disease at presentation and a worse outcome when compared to the first generation [9]. The best predictors of prognosis are the number of family members affected and metastasis at presentation, both of which increase mortality [10]. However, one study suggested that if treated early, FNMTC does not decrease life expectancy of patients [11].

Total thyroidectomy was performed for all patients with additional neck dissection for Patients 2 and 3. Furthermore, all three patients were given RAI^131^. This signifies the role of aggressive treatment in the face of FNMTC. Sippel *et al*. recommend this approach followed by RAI and thyroid hormone suppression therapy to prevent recurrence and decrease mortality [3].

## Conclusions

As specific gene testing is not available, identification of cases of FNMTC relies on a good family history and detailed pedigree analysis. In cases where clinical data suggest the presence of FNMTC, ultrasound should be used for the screening of close relatives for earlier diagnosis and better outcomes. Since FNMTC is known to be particularly aggressive, patients should be a treated with total thyroidectomy and neck dissection and kept under close follow-up with regular evaluations to detect recurrences.

## Abbreviations

ATA, American Thyroid Association; FNA, fine-needle aspiration; FNAC, fine-needle aspiration cytology; FNMTC, familial non-medullary thyroid carcinoma; NMTC, non-medullary thyroid carcinoma; PET, positron emission tomography; RAI^131^, radioactive iodine^131^; SUV, standardized uptake value; T_3_, triiodothyronine; T_4_, thyroxine; TPO, thyroid peroxidase; TSH, thyroid-stimulating hormone
